# Transactions of the Medical Society of the State of New York

**Published:** 1846-06

**Authors:** 


### ARTICLE VIII.

*Transactions of the Medical Society of the State of New York.* Vol. VI. Part III.

The published transactions of this Society, now before us, present as usual, a collection of most interesting and instructive matter.

The Address of Dr. Thos. Hun, on *phlebitis*, delivered before the Albany Medical Society, expresses sound views,—gives evidence that the writer is thoroughly acquainted with his subject, and is in every respect, very creditable to the author. We shall transfer a part or the whole of it—space permitting—to the pages of this number, (the first of the selections) believing that it is one which will be both acceptable and instructive to our readers, especially as it treats of a disease more common than it was formerly supposed to be, and upon which little has as yet been written.

In the next article, Dr. N. S. Davis, under the head of "Observations on an *obscure point* in pathology," gives a few interesting cases, and makes some judicious remarks upon that class of diseases in which, without any appreciable organic lesion, numerous distressing symptoms frequently occur,—such as pain and distressed feeling in the region of the heart and stomach; sensibility to the heart's action; a sense of fullness in the pit of the stomach, and a strong desire for and relief from firm outward pressure,—symptoms, in fact, generally termed *nervous*, a word like that of *bilious*, as remarks the author, "about as often used to cover up professional ignorance, and satisfy the patient's curiosity, as it is to express any well defined idea of disease."

Dr. Davis concludes with the suggestion, that all of these distressing symptoms may depend upon a morbid condition of the system of organic nerves.

Next in order, is an Address, delivered before the Monroe

County Medical Society, by E. W. Armstrong, M. D., President of the Society, in which the claims of our profession to public confidence are clearly and eloquently presented.

After this comes an analysis of the testimony on the trial of A. Cornell, for murder, in which insanity was pled in defence.

Following this interesting analysis, is the Appendix, in which is presented an abstract of the proceedings of the Society. W. B. H.

